# Effectiveness of current hygiene practices on minimization of *Listeria monocytogenes* in different mushroom production‐related environments

**DOI:** 10.1002/fsn3.1629

**Published:** 2020-05-20

**Authors:** Vincenzo Pennone, Kenneth Lyonel Dygico, Aidan Coffey, Cormac G.M. Gahan, Helen Grogan, Olivia McAuliffe, Catherine M. Burgess, Kieran Jordan

**Affiliations:** ^1^ Teagasc research centre Moorepark Ireland; ^2^ Cork Institute of Technology Bishopstown Ireland; ^3^ Teagasc research centre Ashtown Ireland; ^4^ University College Cork Cork Ireland

**Keywords:** casing, growing, hygiene, *Listeria monocytogenes*, mushroom production, substrate

## Abstract

**Background:**

The commercial production of *Agaricus bisporus* is a three stage process: 1) production of compost, also called “substrate”; 2) production of casing soil; and 3) production of the mushrooms. Hygiene practices are undertaken at each stage: pasteurization of the substrate, hygiene practices applied during the production of casing soil, postharvest steam cookout, and disinfection at the mushroom production facilities. However, despite these measures, foodborne pathogens, including *Listeria monocytogenes,* are reported in the mushroom production environment. In this work, the presence of *L. monocytogenes* was evaluated before and after the application of hygiene practices at each stage of mushroom production with swabs, samples of substrate, casing, and spent mushroom growing substrates.

**Results:**

*L. monocytogenes* was not detected in any casing or substrate sample by enumeration according to BS EN ISO 11290‐2:1998. Analysis of the substrate showed that *L. monocytogenes* was absent in 10 Phase II samples following pasteurization, but was then present in 40% of 10 Phase III samples. At the casing production facility, 31% of 59 samples were positive. Hygiene improvements were applied, and after four sampling occasions, 22% of 37 samples were positive, but no statistically significant difference was observed (*p* > .05). At mushroom production facilities, the steam cookout process inactivated *L. monocytogenes* in the spent growth substrate, but 13% of 15 floor swabs at Company 1 and 19% of 16 floor swabs at Company 2, taken after disinfection, were positive.

**Conclusion:**

These results showed the possibility of *L. monocytogenes* recontamination of Phase III substrate, cross‐contamination at the casing production stage and possible survival after postharvest hygiene practices at the mushroom growing facilities. This information will support the development of targeted measures to minimize *L. monocytogenes* in the mushroom industry.

## Graphical abstract

During the mushroom production, the current hygiene practices are not completely efficient in *Listeria monocytogenes* removal. Here, we investigate the effectiveness of hygiene practices in critical points throughout the mushroom production chain.

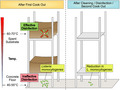

**Highlights**

- In the mushroom production sector, the hygiene practices were not always effective against *Listeria monocytogenes*;
- *L. monocytogenes* can be present in the prepared substrates, such as casing and growth substrate, below the enumeration limit;
- During the steam cookout process, *L. monocytogenes* can survive on floors where the temperature only reached 40–55°C.
- *L. monocytogenes* biofilm formation, disinfectant resistance, and persistence characteristics may limit its effective inactivation.

## 1 INTRODUCTION

*Listeria monocytogenes* is a foodborne pathogen of major concern in the food industry, especially in the ready to eat (RTE) foods sector (Farber & Peterkin, 1991). The ability of this organism to cause disease, and its presence in food leading to product recalls, is a threat to public health and for the food production industry (Buchanan, Gorris, Hayman, Jackson, & Whiting, 2017). *Agaricus bisporus* is a widely distributed industrially produced mushroom, considered RTE due to its use in raw salads (FSAI, 2006). *L. monocytogenes* has been previously found in the mushroom production environment, especially on wet floors, slicers, and packaging areas (Murugesan, Kucerova, Knabel, & Laborde, 2015). No outbreaks of listeriosis have been recorded due to contamination of cultivated mushrooms, but there have been several product recalls in recent years (EU, RASFF; FSAI, 2006), raising awareness among the mushroom producers of this potential hazard.

The production of cultivated mushrooms involves three distinct stages: mushroom growth substrate (often called “substrate” or “compost”) production, mushroom casing production, and mushroom growing. Substrate production consists of three phases. Firstly, manure and prewet straw are mixed and stored for up to one week, to start the process. The mix is then moved into bunkers for up to 7 days in a process called “Phase I,” where the microbial aerobic digestion starts and the temperatures can reach 80°C (Zhang et al., 2014). The process continues with a pasteurization and conditioning step (Phase II); an initial heat treatment at 60°C for up to 14 hr to inactivate any unwanted microbiota, followed by a conditioning step at 45°C for 4–6 days to create a substrate suitable for mushroom colonization (Straatsma et al., 2000). After pasteurization and conditioning, the substrate is inoculated with *A. bisporus* mycelium and incubated aerobically at 25°C for 16–19 days, before being delivered to the mushroom growing facilities as Phase III substrate (Vos, 2017). In stage 2, casing soil production, which, in Ireland, consists of mechanically mixing peat and lime, is followed by delivery in bags or bulk to the mushroom farm. In order to preserve the natural microbiota of the raw materials that influence mushroom development, there are no antimicrobial steps during the production of casing soil (Cai et al., 2009; McGee, 2018; Siyoum, Surridge, & Korsten, 2010). However, hygiene measures to reduce cross‐contamination, such as the use of disinfectants and the building of physical barriers between storage areas, are implemented in production facilities. The third stage of the process is mushroom growing at dedicated mushroom production facilities. Phase III substrate is delivered to the facilities and filled onto shelves, and a layer of casing soil is then placed on top of it. The natural microflora of the casing stimulates the growth of the mushroom fruiting bodies (McGee, 2018). The growing substrates are incubated at 22–25°C for 7 days to encourage mushroom colonization of the casing soil. By modifying the temperature, humidity, and CO_2_ content of the growing room, the fruiting body of the mushroom is stimulated and formed (Kertesz & Thai, 2018). Mushroom harvesting generally lasts for three weeks, with three flushes (harvesting times) per crop. While the cleaning and disinfection procedures are not standardized across mushroom growing facilities, good agricultural practices are applied. In general, there is an initial steam‐treatment at 60–70°C in the growing room after crop harvesting. This process introduces live steam into the growing room with the aim of achieving a temperature of 60–70°C in the substrate for 6–8 hr. The whole process takes 18–20 hr and is referred to as “steam cookout.” Its aim is to inactivate all the pathogens and pests that might be present in the growing substrates and growing area, although the temperatures reached on the concrete floors are likely to be lower than the 60–70°C reached in the substrate. Subsequently, the growing room is emptied, cleaned with a power‐hose, and sprayed with a disinfectant such as sodium hypochlorite. This may be followed by a second steam‐treatment at 60–70°C for 3–6 hr, to ensure that all the surfaces are disinfected before a new crop is started.

The aim of this study was to assess the impact of the hygiene practices applied across the different stages of the mushroom production process on the occurrence of *L. monocytogenes*.

## 2 MATERIALS AND METHODS

### 2.1 Bacterial strains used in this study

The *L. monocytogenes* strain Scott A (reference strain) and *L. monocytogenes* strain 2081, a mushroom industry isolate previously shown to be a strong biofilm former (Dygico, Gahan, Grogan, & Burgess, 2019), were used as control strains. The *L. monocytogenes* strains 3,050, 3,051, 3,101, 3,102, and 3,104 were isolated from floor swabs during this study, after cookout, during the sampling of mushroom production facilities. All the strains were stored on Protect beads (Technical Service Consultants Ltd., UK) and 50% glycerol at −80°C and resuscitated by streaking a bead onto Tryptone Soya Agar (TSA; Oxoid, UK) and incubating at 37°C for 24 hr.

### 2.2 Sampling plan for each stage of mushroom production

**Figure 1:**
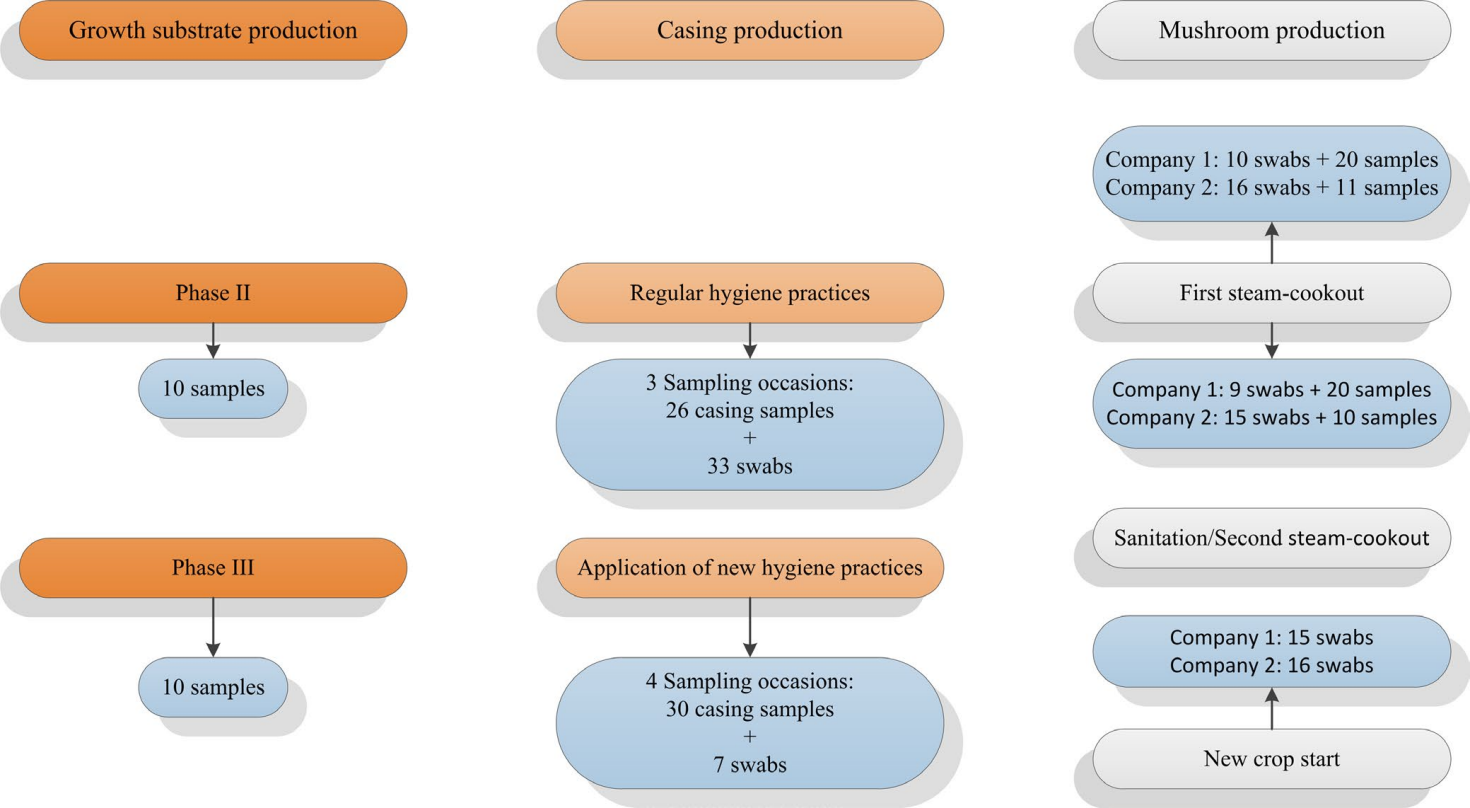
Schematic representation of the sampling plan. In the growth substrate production unit, samples were analyzed after Phase II and Phase III processes. In the casing production unit, samples and swabs were analyzed before and after the application of new hygiene practices. In the mushroom production facilities, samples and swabs were analyzed before and after the first cookout and swabs were analyzed before the start of a new mushroom crop

One substrate producer, one casing producer, and two mushroom producers agreed to participate in the study of the assessment on the effectiveness of hygiene practices in the mushroom production chain. The sampling plan is shown in Figure 1.

At the substrate production facility, samples were taken after Phase II and Phase III. Samples of Phase II and Phase III substrate (approx. 50 g each sample, in a sterile plastic bag) were shipped from the substrate producer via express courier, and the samples were analyzed within 24 hr of being taken.

The casing production facility was visited on seven occasions. Three visits were undertaken at the start of the study, to assess the presence of *L. monocytogenes* at the facility, and four visits after the company implemented changes to hygiene processes. Initially, hygiene practices consisted of washing the conveyor belts with disinfectants every one or two years, and huge quantities of raw material stocks were kept in outdoor bays, until replacement. At each visit, swabs were taken from the conveyor belts in the general facility, floors, and loaders, while casing samples were taken from the storage bays and conveyor belts in the storage bays.

**Figure 2:**
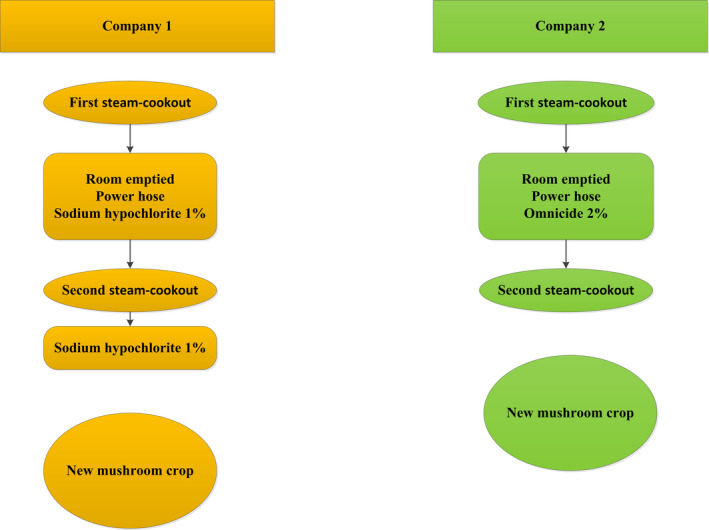
Postharvest hygiene procedures applied by the two mushroom producers involved in this study

Samples were taken at two different mushroom growing facilities. The two mushroom producers used slightly different hygiene processes between mushroom crops (Figure 2). In Company 1, following the first steam cookout process, where the aim was to increase the substrate temperature to between 60 and 70°C for 12 hr, the room was emptied, cleaned with a power‐hose and sodium hypochlorite (1%) was applied to all the surfaces. A second steam cookout raised the air temperature of the empty growing room to 60–70°C for 3 hr, followed by another treatment with sodium hypochlorite. In Company 2, the first steam cookout process was similar to that of Company 1, with the temperature of the substrate raised to 60–70°C for 12 hr. The room was then emptied, cleaned with a power‐hose and disinfected with a quaternary ammonium compound‐based product (Omnicide 2%). A second steam cookout of the empty room at 60–70°C for 12 hr was undertaken, before starting a new mushroom crop. The samples taken were a mixture of casing, substrate, and mushroom residues from the growing shelves, at the end of mushroom production, before and after the cookout process, and before starting a new mushroom crop.

### 2.3 Temperature recording

In order to determine whether the expected temperatures were reached in the mushroom growing rooms during cookout, temperature probes (LS Technology) were placed in the substrate on the shelves and on the floor during cookout.

### 2.4 *L. monocytogenes* detection and enumeration

All swabs were tested for the presence of *L. monocytogenes*, as described by the BS EN ISO 11290–1:1997 + A1:2004. Samples of Phase II and Phase III substrate and casing samples (from substrate and casing producers) as well as samples of spent substrate taken at the end of the mushroom crop, before, and after cookout, were tested for the presence and enumeration of *L. monocytogenes*, as described by the BS EN ISO 11290‐1 and 11290‐2:1998. Presumptive *L. monocytogenes* isolates, blue‐green colonies with an opaque halo on Agar *Listeria* acc. to Ottaviani Agosti (ALOA, BioMérieux, France), were grown in Brain Heart Infusion (BHI) broth (Merck, Darmstadt, Germany) and stored in cryovials containing 20% glycerol at −20°C for further characterization.

### 2.5 Confirmation of isolates as *L. monocytogenes*

To confirm the isolates as *L. monocytogenes*, polymerase chain reaction (PCR) was performed according to Ryu et al., (2013). The isolates were serogrouped by PCR according to Doumith, Buchrieser, Glaser, Jacquet, & Martin, (2004) and Pulsed Field Gel Electrophoresis was performed according to the PulseNet protocol (Anonymous, 2013). BioNumerics 7.6 (Applied Maths, Sint‐Martens‐Latem, Belgium) was used for comparison of the genetic profiles (pulsotypes).

### 2.6 Heat inactivation studies

*L. monocytogenes* strains 3,050, 3,051, 3,101, 3,102, and 3,104, isolated postcookout, along with strains Scott A and 2081, were tested in heat inactivation studies. The inocula were prepared from overnight cultures of each *L. monocytogenes* strain grown in BHI broth. The cultures were diluted to log_10_ 5 CFU/ml, centrifuged at 5,000 x g for 10 min, and resuspended in an equal volume of Maximum Recovery Diluent (MRD). The heat inactivation experiments were conducted with a coil apparatus that was submerged in a temperature controlled water bath (TE‐10D tempette), operated at 50°C, 60°C, and 65°C. The inoculum was introduced into the coil apparatus using a 10‐ml syringe and 400 µl samples were collected into vials filled with 1.6 ml of MRD at consistent time intervals, then immediately cooled on ice. The heated and cooled samples were then enumerated by serial dilution and spread plating 100 µl from each dilution on to TSA plates in duplicate. The plates were then incubated at 37°C for 24 hr. Each experiment for each strain tested at each temperature was independently repeated four times.

### 2.7 Capacity of the *L. monocytogenes* isolates to form biofilm

Similar to the heat inactivation experiment, the same strains (*n* = 7) were tested for their biofilm‐forming potential based on the method described by Bolocan et al., (2017), with minor modifications. Briefly, liquid cultures of each strain in BHI broth supplemented with 0.6% yeast extract (BHIYE; Oxoid, UK) and were grown overnight at 37°C. The cell concentration of the overnight cultures was adjusted to approximately log_10_ 7 CFU/ml, centrifuged at 5,000 × g for 7 min at 4°C and then resuspended in fresh BHIYE or BHIYE diluted 1:20 in deionized water (dBHIYE). Two hundred microliter aliquots of freshly prepared liquid culture were inoculated into three wells of a sterile round‐bottomed polystyrene tissue culture plate (Corning, NY, USA). *L. monocytogenes* strain 2081 was included as a strong biofilm‐forming control and sterile media (BHIYE and dBHIYE) as negative controls. The microtiter plates were then incubated statically in aerobic conditions for 72 hr at 18°C and 25°C, chosen to reflect the temperatures at different growth stages of the mushroom crop. The wells were then washed with a 200 µl aliquot of phosphate‐buffered saline (PBS) three times to remove unattached cells. The remaining cells were then fixed with 95% methanol for 15 min and allowed to air dry. The fixed cells were then stained with 150 µl of 0.2% w/v crystal violet solution (Sigma‐Aldrich) for 15 min, and then, the excess stain was rinsed off under gently running tap water. The stain was then resolubilized using 35% acetic acid and the absorbance measured at 595 nm using a Multiskan FC Microplate Photometer (Thermo Fisher Scientific). Each experiment was then repeated three times. The results were interpreted based on the formula of Stepanovic, Vukovic, Dakic, Savic, & Svabic‐Vlahovic, (2000). The OD cutoff for the negative control was calculated by using the mean OD of all negative control wells plus three standard deviations (OD_NC_). The strains were then categorized as weak (OD_NC_ < OD ≤2 × OD_NC_), moderate (2 × OD_NC_ <OD ≤ 4 × OD_NC_), or strong (4 × OD_NC_ <OD) biofilm formers.

### 2.8 Statistical analysis

The data obtained were processed with SPSS (version 24.0, SPSS Inc., Chicago IL, USA) to determine whether there was a significant difference between the prevalence of *L. monocytogenes* before and after the application of hygiene practices. Briefly, the data were analyzed for normality distribution with descriptive statistics and, based on the results, parametric or nonparametric tests (ANOVA and Kruskal–Wallis, respectively) were performed.

For the heat inactivation studies, the *D‐*values for each strain at each temperature tested were calculated by obtaining the slope from plots of log_10_ CFU/ml against time and then using the negative reciprocal of the slope from each treatment. One‐way ANOVA was used to compare the *D‐*values for each strain, followed by the Tukey test to determine significant differences between the means (*p* ≤ .05). These tests were also performed using SPSS software.

## 3 RESULTS

All the samples analyzed contained < 10 cfu/g of *L. monocytogenes* as determined by direct plating.

### 3.1 Occurrence of *L. monocytogenes* during substrate production

Ten samples of Phase II compost and 10 samples of Phase III compost, taken at a substrate production facility, were tested for the occurrence of *L. monocytogenes*. All Phase II substrate samples, obtained immediately after pasteurization, showed the absence of *L. monocytogenes*. In contrast, 4 out the 10 samples of Phase III substrate were determined to be positive for *L. monocytogenes*, an occurrence of 40%, indicating a significant difference in the occurrence of *L. monocytogenes* in Phase II and Phase III substrates (*p* < .05,).

### 3.2 Occurrence of *L. monocytogenes* during casing production

The casing production site was initially sampled on three separate occasions. From these samplings, a total of 26 casing samples and 33 swabs were obtained. An overall *L. monocytogenes* occurrence of 31% (18 positive samples) was found, with 9 casing samples positive (34%) and 9 swabs positive (27%). In order to attempt to improve the situation with regard to *L. monocytogenes* occurrence, a number of corrective actions were recommended to the casing producer and implemented. The recommendations included washing procedures for the conveyor belts and transport lorries, the introduction of pools for boot disinfection at the entrance and the exit of all areas, and a decrease in the amount of raw materials in stock, refilling the bays more frequently. After six months, the suggested procedures were implemented and, in addition, structural works were undertaken to build a new structure that contained more storage bays indoors, rather than outdoors.

Following implementation of the corrective actions, a total of 37 samples were taken of which 8 were positive (22%), with no significant difference observed before and after corrective actions (*p* > .05). Looking at the sample types individually, 9 out of 26 casing samples were positive (34%) before the corrective actions and 6 out of 30 (20%) positive after the corrective actions. The swabs were 9 out of 33 (27%) positive before and 2 out of 7 (29%) positive after the corrective actions. After the hygiene corrective actions were implemented, the first batch of casing samples showed an absence of *L. monocytogenes*, but three months later, the occurrence in the casing was 20%.

### 3.3 The effectiveness of steam cookout at mushroom growing facilities

**Figure 3:**
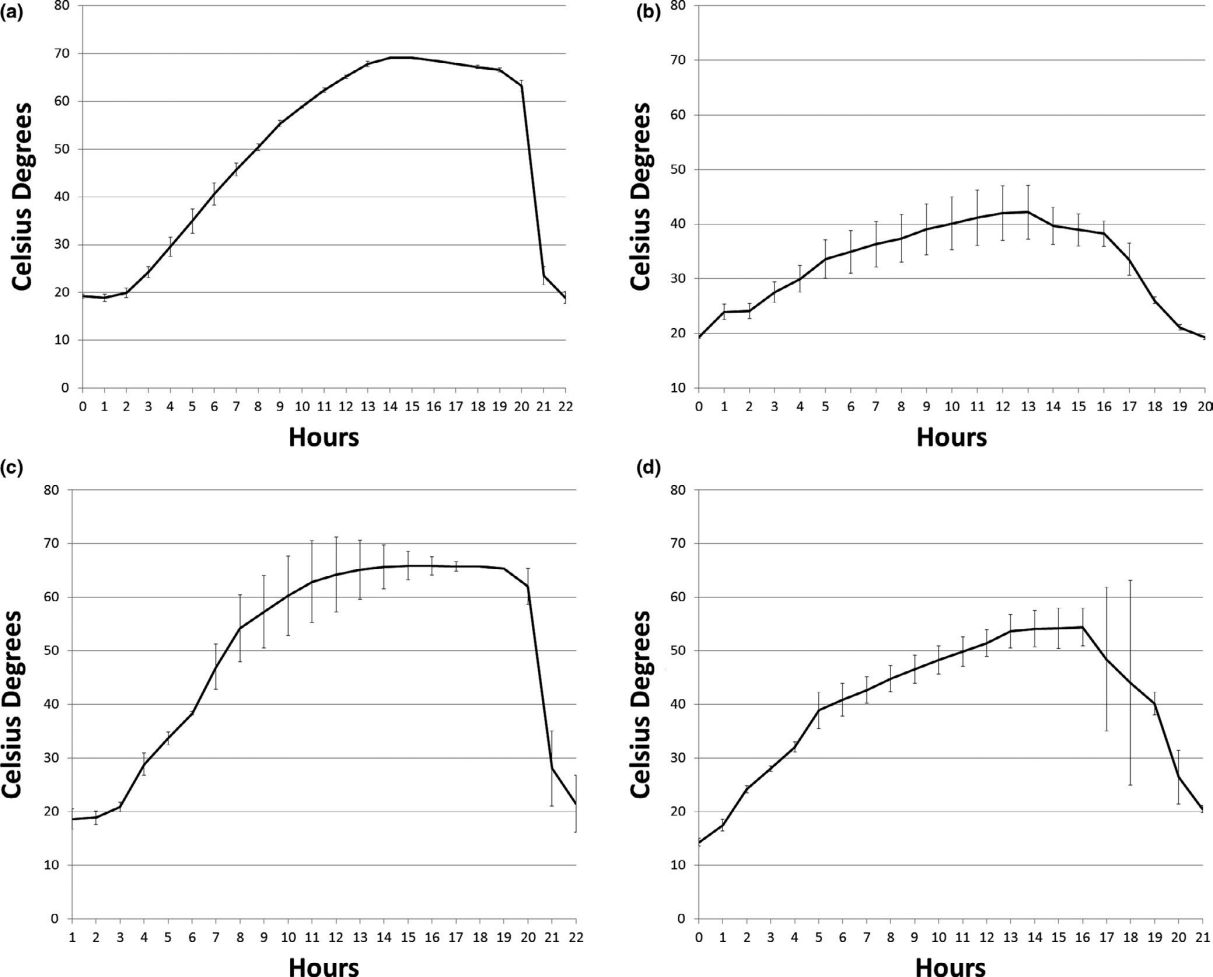
Temperatures recorded during the first steam cookout in Company 1 (a, substrate temperature on shelves and b, floor temperature) and Company 2 (c, substrate temperature on shelves and d, floor temperature). Each point is the average of three measurements, and the standard deviation is represented by the error bars

The mushroom growing facilities were sampled before and after the first cookout and again, after the second cookout and sanitization (Figure 2). During the first cookout process at Company 1, the temperature reached almost 70°C for 10 hr in the spent growth substrates on the shelves, but less than 50°C on the floor (Figure 3a and 3b).

Before cookout in Company 1, the incidence of *L. monocytogenes* was 50% on average, with 100% of the floor swabs and 25% of the spent substrate samples positive (Table 1). After the first cookout, there was an overall presence of *L. monocytogenes* of 17%, with 56% of the floor swabs positive and no positives in the spent substrates. Floor swabs taken after the entire process of sanitation, just before the start of a new crop, showed 13% were positive for *L. monocytogenes*.

**Table 1:** The effect of the steam cookout and sanitation in Company 1

Company 1	Before (%)	After (%)	Final (%)
Floor swabs total	10	9	15
Floor swabs positives	10 (100**%**)	5 (56%)	2 (13%)
Substrate samples total	20	20	–
Substrate samples positives	5 (25%)	0 (0%)	–
Total (all)	30	29	15
Total positives	15 (50%)	5 (17%)	2 (13%)

Note

The table shows the occurrence of *L. monocytogenes* in floor swabs and substrate samples before the first steam cookout (“Before” column), after the first steam cookout (“After” column), and before starting a new mushroom crop (“Final” column).

During the first cookout process at Company 2, the temperature also reached almost 70°C for 10 hr in the spent growth substrates on the shelves and between 50 and 60°C on the floor for 3 hr (Figure 3c and 3d). Before cookout, the incidence of *L. monocytogenes* was 63% on average, with 75% of the floor swabs and 45% of the spent substrates samples positive (Table 2). After the first cookout, there was an overall presence of *L. monocytogenes* of 40%, with 67% of the floor swabs positive and no positives in the spent substrates. Floor swabs taken after the second cookout, just before the start of a new crop, showed that 19% were positive for *L. monocytogenes*.

**Table 2:** Effect of the cookout and sanitation in Company 2.

Company 2	Before (%)	After (%)	Final (%)
Floor swabs total	16	15	16
Floor swabs positives	12 (75%)	10 (67%)	3 (19%)
Substrate samples total	11	10	–
Substrate samples positives	5 (45%)	0 (0%)	–
Total (all)	27	25	16
Total positives	17 (63%)	10 (40%)	3 (19%)

Note

The table shows the occurrence of *L. monocytogenes* before the first cookout (“Before” column), after the first cookout (“After” column), and before starting a new mushroom crop (“Final” column).

### 3.4 Initial characterization of the isolates

Sixty‐one isolates were recovered from the whole sampling period, 26 from Company 1 and 29 from Company 2, 4 from the casing producer, and 2 from the substrate producer. All the isolates were confirmed as *L. monocytogenes* by PCR. The isolates were serogrouped by PCR, with 18 isolates being serogroup 1/2a‐3a, 16 isolates being serogroup 4b‐4d‐4e, and 27 isolates being serogroup 1/2b‐3b‐7.

### 3.5 Pulsed Field Gel Electrophoresis analysis of the isolates

**Figure 4:**
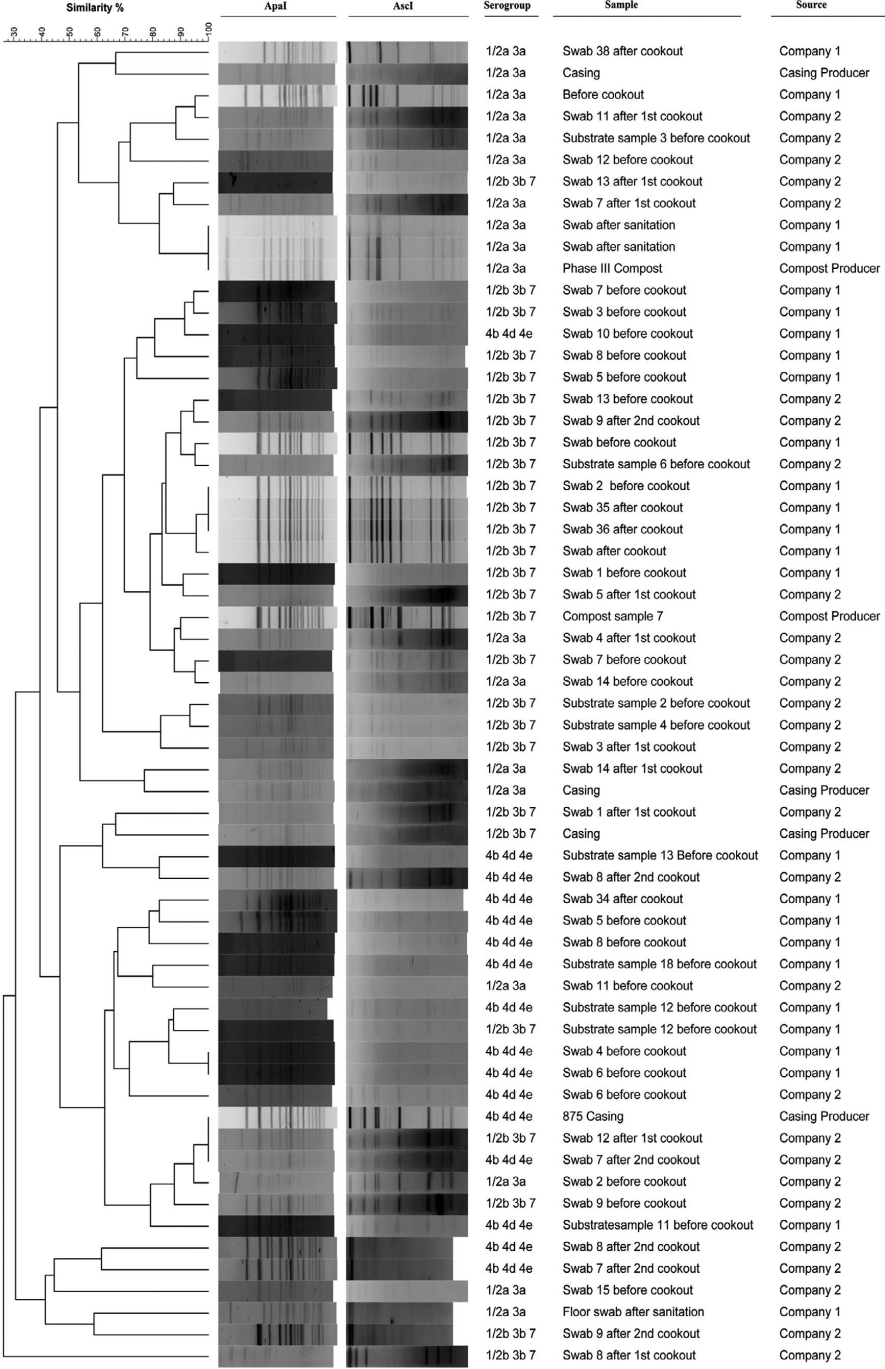
Dendrogram obtained from the PFGE analysis of 61 isolates of *L. monocytogenes* from samples taken at two mushroom production facilities, one casing and one Phase III substrate producers. The fingerprint files were analyzed with BioNumerics, and 36 distinct pulsotypes were identified, with a cutoff of 86% similarity

**Figure 5:**
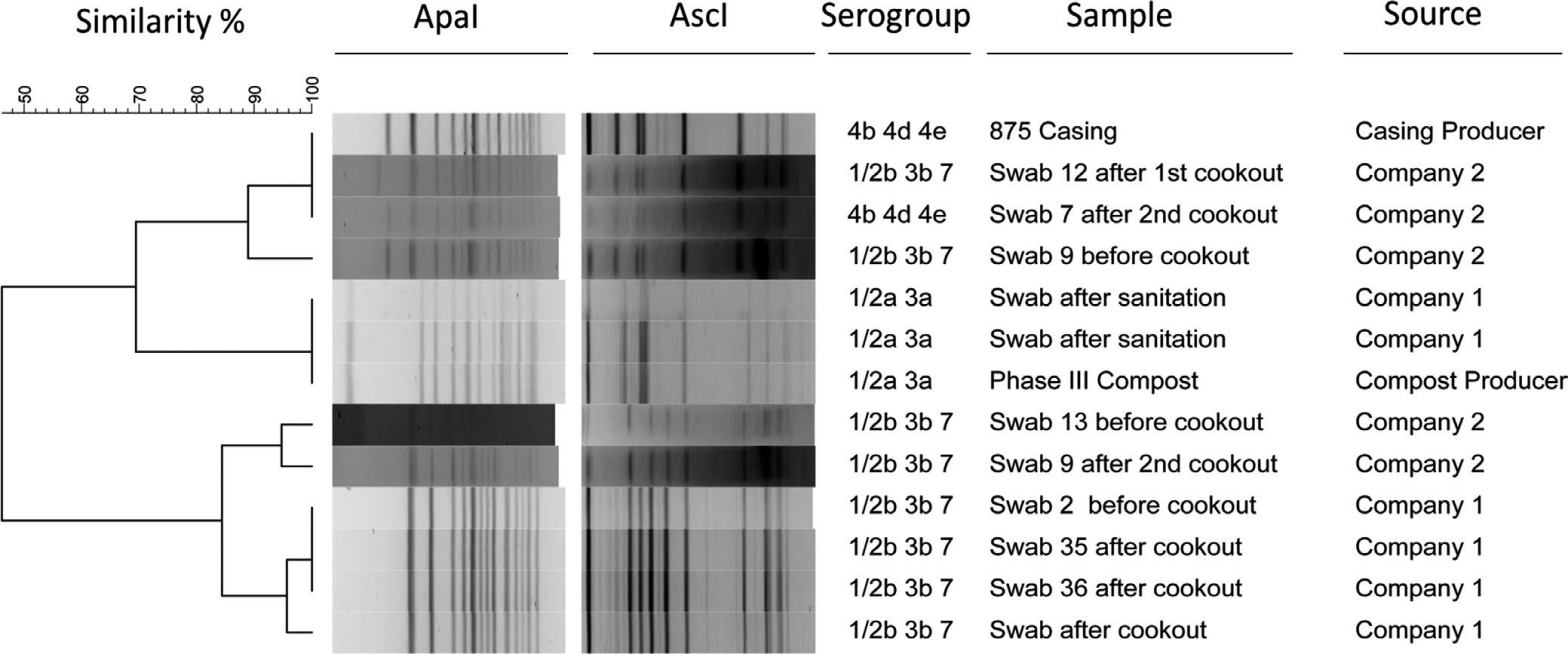
Dendrogram showing the four pulsotypes where similarities were found between isolates obtained before/after cookout and after sanitation. Some of Company 1 and Company 2 isolates showed also similarities with the casing and substrate producers, respectively (cutoff at 86% similarity)

**Figure 6:**
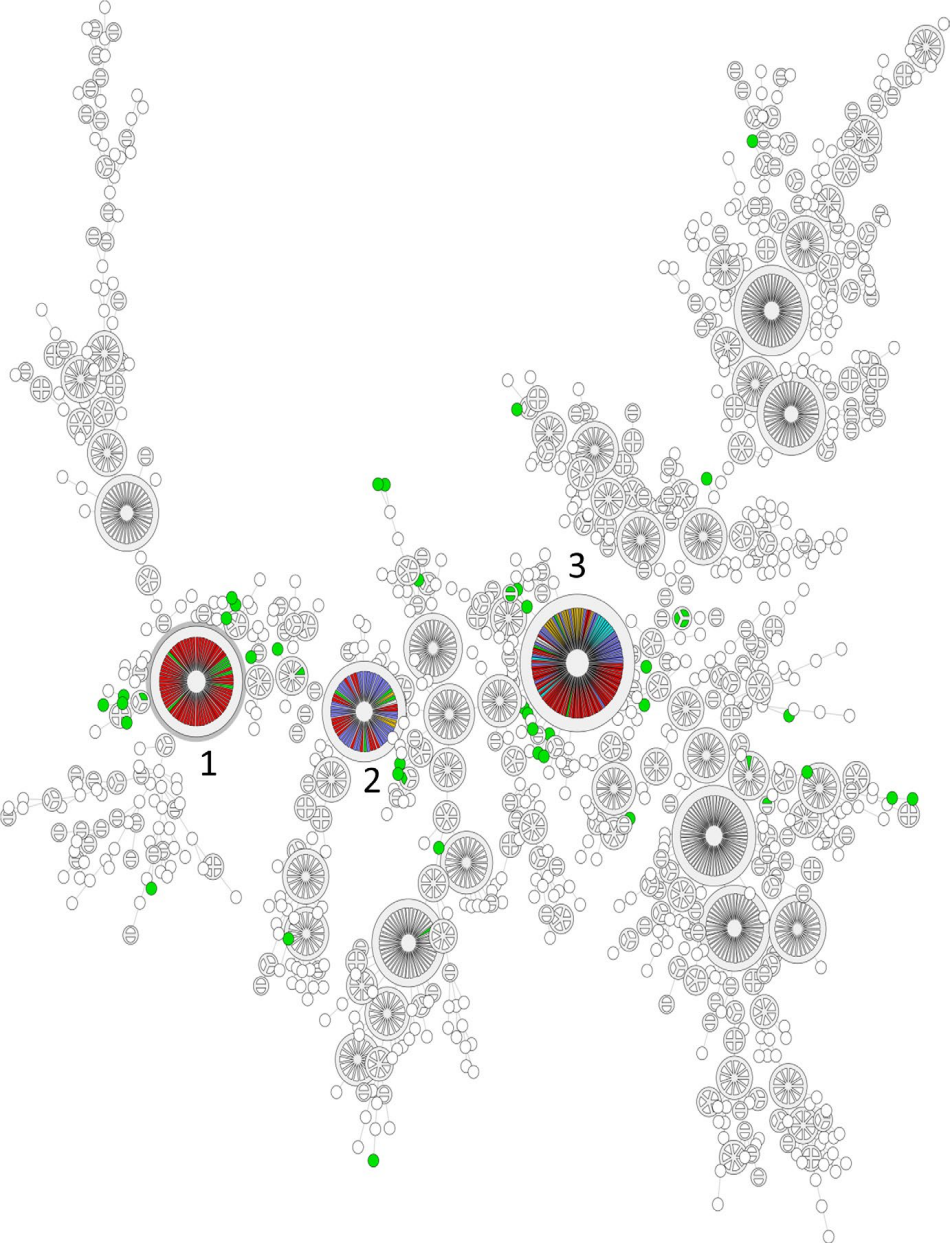
Minimum Spanning Tree, built with BioNumerics, based on the similarity matrix of the isolates obtained in this study (green) and the entire database of 3,000 isolates of *L. monocytogenes* obtained from different food production sectors (red, mushroom production; purple, cheese production and light blue, other sectors) and clinical isolates (yellow). Three big clusters have been highlighted (numbers 1, 2, and 3), formed by a mixture of all the different categories, including isolates from the current study

Sixty‐one isolates in total were characterized by PFGE. The pulsotypes (*n* = 36) identified are shown in Figure 4. Four pulsotypes included isolates obtained from the same company before and after the cookout process and before and after the sanitation procedure (Figure 5). Two of those profiles showed similarity with casing and Phase III substrate isolates (Figure 5). All the 61 isolates were compared with a full database of about 3,000 *L. monocytogenes* isolates, obtained from different food sectors and clinical isolates, with the results shown in a minimum spanning tree (Figure 6). Three large clusters, in particular, were identified and highlighted, where a mixture of isolates obtained from different food sectors and clinical isolates shared the same pulsotype, including isolates from the current study (Figure 6).

### 3.6 Heat tolerance of the isolates

The *D‐*values for heat resistance obtained for all of the *L. monocytogenes* strains tested is shown in Table 3. While there were some minor statistical differences between the strains, none of the cookout survivors showed excessive survival of heat treatment. The cookout survivor strain 3,104 was found to have a significantly higher *D*_50_‐value (550.4 min) compared to the other strains tested (*p* < .05). Scott A was also found to have a significantly higher *D*_50_‐value (343 min) than the mushroom industry isolates (*p* < .05), except strain 3,104 (*p* > .05). No significant differences (*p* > .05) were found in the *D*_50_‐values between strain 2081, a mushroom industry persistent isolate, and the remaining cookout survivors. There was no significant difference found between the *D*_60_‐values of all strains (*p* > .05). At 65°C, the cookout survivor strain 3,102 was found to be the most heat tolerant with a significantly higher *D*_65_‐value than strains 3,050, 3,104, and 2081 (*p* < .05).

**Table 3:** Heat tolerance of *L. monocytogenes* strains isolated after cookout

Temperature	Strains	*D‐*value (min)	
		Mean^a^	SE
50°C	3,050	144.1^C^	11.0
	3,051	161.7^C^	17.6
	3,101	175.8^C^	18.3
	3,102	206.8^C^	46.0
	3,104	550.4^A^	25.5
	2081^b^	177.9^C^	23.0
	Scott A^b^	343.0^B^	31.8
60°C	3,050	1.3^A^	0.1
	3,051	1.3^A^	0.0
	3,101	1.5^A^	0.1
	3,102	1.2^A^	0.1
	3,104	1.5^A^	0.1
	2081	1.4^A^	0.1
	Scott A	1.2^A^	0.1
65°C	3,050	0.6^B^	0.0
	3,051	0.7^AB^	0.0
	3,101	0.7^AB^	0.0
	3,102	0.9^A^	0.0
	3,104	0.7^B^	0.0
	2081	0.6^B^	0.0
	Scott A	0.7^AB^	0.0

Note

tested are significantly different (*p* < .05).

^a^ Values with different letters (A–C) within each temperature.

^b^ control strains.

### 3.7 Biofilm formation ability of the isolates

**Figure 7:**
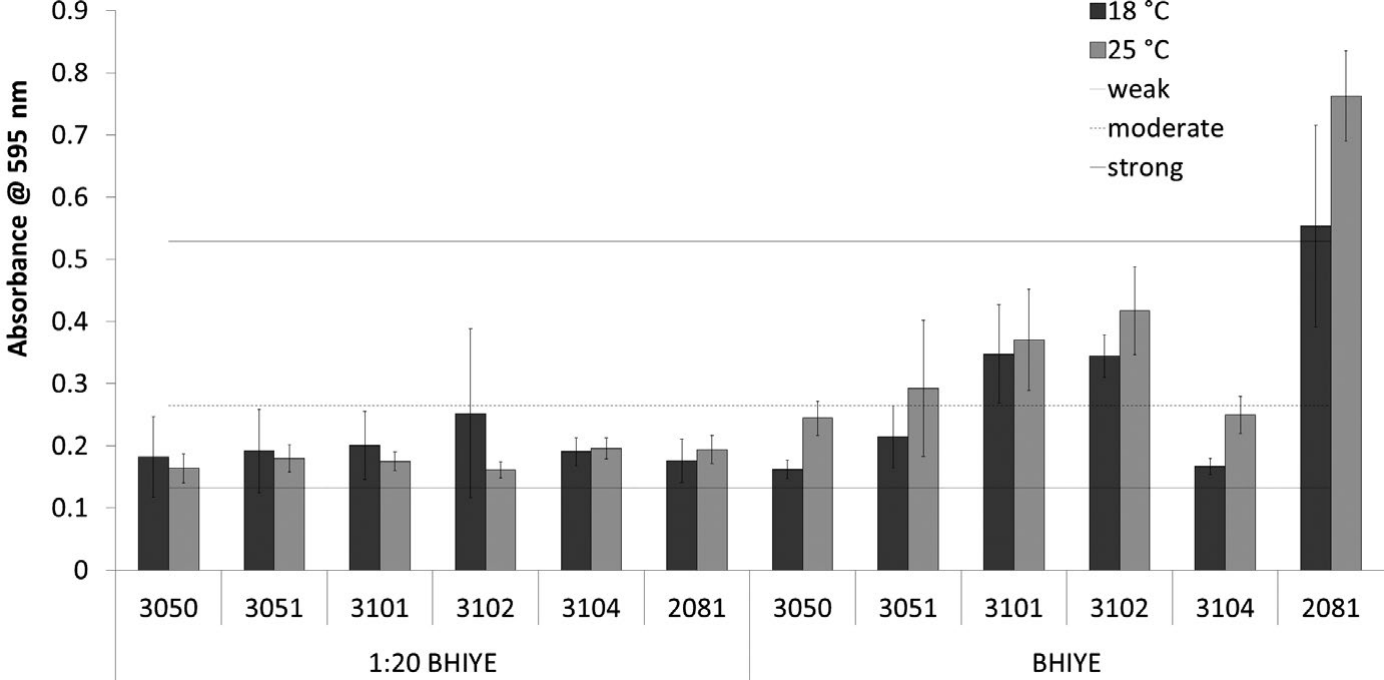
Mean biofilm formation, including standard deviation, of each *L. monocytogenes* strain at different temperatures. The biofilm‐forming thresholds were set at 0.132 for weak, 0.265 for moderate, and 0.529 for strong according to the formula described by Stepanović *et al.* (2000)

All of the strains isolated after the final cookout/disinfection stage at the two mushroom production facilities were tested for their ability to form biofilm using the crystal violet assay. As shown in Figure 7, the biofilm formation ability of the strains was significantly less (*p* < .05) than the control strain on polystyrene microtiter plates at industry relevant temperatures. In BHIYE at 18°C, strains 3,050, 3,051, and 3,104 all formed weak biofilms, strains 3,101 and 3,102 formed moderate biofilm, while the control strain 2081 formed strong biofilm. At 25°C, the biofilms for each strain were categorized similarly, except for strain 3,051 which formed moderate biofilm. In 1:20 BHIYE (nutrient‐poor condition), all strains formed weak biofilm at both temperatures tested.

## 4 DISCUSSION

The results of this study indicate that the current hygiene practices employed across the three distinct stages of the mushroom production process do not appear to eliminate *L. monocytogenes* completely from the mushroom production environment, and if they do, recontamination can occur Weil, Cutter, Beelman, & LaBorde, (2013) conducted a study to establish the efficiency of substrate pasteurization on inactivation of various pathogens, including *L. monocytogenes*. The study showed that only two hours at 60°C were necessary to completely inactivate *L. monocytogenes* in Phase II substrate. In this study, none of the samples were positive for *L. monocytogenes* after pasteurization of the substrate. Although the occurrence of *L. monocytogenes* was not shown before pasteurization, due to the nature of the raw material (e.g., chicken manure and straw) it is likely that they were present. Some positives were found in the Phase III samples, indicating potential recontamination on site. Although no samples of Phase I were analyzed in this study, *L. monocytogenes* has been detected in Phase I substrate in past studies (Viswanath et al., 2013). It is acknowledged that there are some limitations to this study as the sample size for this aspect of the study was small, as only one substrate producer participated in the study. Furthermore, after Phase II, the procedure for inoculating the substrate with mushroom spawn is very strict regarding sterility and biosecurity and obtaining samples during this delicate step was not feasible.

However, the results shown suggest that the manipulation of Phase II substrate is a critical point, where cross‐contamination could occur, leading to the occurrence of *L*. *monocytogenes* in Phase III substrate. Such contamination could occur during the transportation and spawning processes, for example. Additionally, the cleaning and disinfection of the incubation tunnels for the Phase III substrate may not be adequate and could allow cross‐contamination to occur. More targeted studies are required to clarify the potential routes of cross‐contamination during production of Phase III substrate before it is delivered to the mushroom growers.

After the first sampling at the casing producer, corrective actions to address the relatively high occurrence of *L*. *monocytogenes* were suggested (as detailed in the results section). Although the implementation of the corrective actions was not monitored over time, it is clear from the results that recontamination of the casing is always a possibility or that there is a background level of *L. monocytogenes* in the raw materials.

Some studies have shown the possibility of *L. monocytogenes* surviving in the casing soil and suggested preharvest treatments for pathogen control on the mushrooms, especially with interventions in the irrigation water (Chikthimmah, 2006). In a casing soil production facility, it is almost impossible to control the spread of *L. monocytogenes*, because of its ubiquitous presence, especially in soils (Weis & Seeliger, 1975). This illustrates that there needs to be a better understanding of the routes of cross‐contamination in the casing production environment and that there needs to be ongoing emphasis on the importance of hygiene measures.

The PFGE profiles obtained in this study showed the presence of the same pulsotypes before, during, and after the hygiene procedures in the mushroom production facilities (Figures 4 and 5). Furthermore, the analysis of pulsotypes showed a possible cross‐contamination scenario from the prepared growth and casing substrates to the mushroom growing facility (Figure 5). Previous studies have already highlighted *L. monocytogenes* persistence and cross‐contamination in the mushroom industry in the Republic of Ireland (Leong et al., 2017; Madden et al., 2018; Pennone, Lehardy, Coffey, McAuliffe, & Jordan, 2018). In this study, similarities have been found between the isolates characterized by PFGE and isolates obtained from other food sectors and with clinical isolates (Figure 6). However, rather than cross‐contamination, the presence of common ubiquitous clones of *L. monocytogenes* could determine the formation of these big clusters with the same pulsotype isolated at different times and in different places with no apparent link (Chenal‐Francisque et al., 2013; Leong et al., 2017). The similarities found with clinical isolates demonstrate that the mushroom isolates obtained have the potential to cause disease, and further study of these isolates is necessary to characterize virulence.

The cookout process used by both the mushroom growing facilities in this study did not totally inactivate *L*. *monocytogenes* on the floor. No isolates were obtained after cookout from the spent casing/substrate mixture on the growing shelves. At these locations, the temperature reached the target value of 60–70°C for several hours and there was inactivation of *L*. *monocytogenes* in all cases. However, isolates were obtained after cookout from the floor samples at both facilities. The temperature of the floor, which only reached 40–55°C (Figure 3), was not sufficient to inactivate *L*. *monocytogenes*. None of the floors at the facilities were insulated, making it difficult to achieve the temperature needed to inactivate *L. monocytogenes*. Particular attention should be given in the construction of new mushroom growing houses to insulation of the floors, to facilitate achieving higher floor temperatures during cookout and ensuring that microbial inactivation is achievable. Alternatively, a steaming operation that targets floors specifically could be considered. The isolates obtained after cookout were not shown to have significantly higher heat tolerance than the control strains, except for isolate 3,104 which had significantly higher tolerance at 50°C. McDermott, Whyte, Brunton, & Bolton (2018) observed similar *D*_50_‐values and *D*_60_‐values from crab isolates of *L. monocytogenes* in TSB with 176 min and 1.4 min, respectively. Interestingly, the *D*_60_‐values of the strains tested in this study had similar results to the low heat tolerant *L. monocytogenes* strains categorized by Shen et al., (2014). Exposure to sublethal heat has been previously shown to increase the heat resistance of *L. monocytogenes* strains and this may have played a role in the high heat resistance observed in strain 3,104 (Linton, Webster, Pierson, Bishop, & Hackney, 1992; Shen et al., 2014). However, further analysis will be required to elucidate this.

In addition to surviving the cookout temperature of the floors, some of the strains that survived also resisted sanitation and a further heat treatment. It is possible that the strains were resistant to the sanitation process, as described in other studies (Møretrø et al., 2017), but it is more likely that they survived in niches where the disinfectant concentration was more diluted (for review see (Carpentier & Cerf, 2011), in areas where the sanitation process was not completed properly, or where biofilms were formed (Lourenco, Machado, & Brito, 2011), although in the tests done there was little evidence for strong biofilm formation. Further characterization of the isolates with regard to disinfectant resistance and biofilm formation would be needed to investigate this. Nonetheless, the results of this study show that steam cookout as it is practiced at these two facilities was not as effective at inactivating *L. monocytogenes* on floors as previously thought. Improvements in the floor disinfection are needed after the cookout procedure in order to achieve complete inactivation of *L*. *monocytogenes* between mushroom crops.

Based on the results of this study, three critical areas for the entry of *L*. *monocytogenes* can be identified in a mushroom growing facility:

‐ *L. monocytogenes* can enter in the facility via prepared substrates, such as casing and growth substrate;
‐ *L. monocytogenes* can survive the steam cookout process on floors where the floor does not reach the required temperature;
‐ Biofilm formation, disinfectant resistance, and persistence characteristics decrease the possibility of successfully removing *L. monocytogenes* from the production environment.

Attention to detail in the application of the current HACCP (hazard analysis critical control point)‐based practices, or alternative approaches are therefore needed to control *L*. *monocytogenes* during mushroom production. Training of staff in awareness of *L*. *monocytogenes* at production facilities and reduction of cross‐contamination may reduce the risk of mushroom contamination. The efficacy of postharvest treatments of whole or sliced mushrooms against *L. monocytogenes* has not been addressed in the current study, but treatment with bacteriophages, various disinfectants and UV light have been studied by Murray, Wu, Aktar, Namvar, & Warriner, (2015). However, issues with efficacy, the quality of the mushrooms, and the applicability of the processes on large scale need to be addressed. A whole chain approach to minimizing *L. monocytogenes* contamination would be of significant benefit to the industry as a whole.

In this study, it was shown that the efficacy of the hygiene practices at the three distinct stages of the mushroom production process (casing, substrate, and mushroom production) are not always effective for the total removal of *L. monocytogenes*. Two issues in particular are related to the presence of *L. monocytogenes;* its occurrence in the prepared substrates on the one hand, and the ineffectiveness of the steam cookout procedure on the floors of mushroom growing units on the other. More in‐depth studies are required to address the issue of cross‐contamination at each level of mushroom production. Consideration should be given to the implementation of structural changes, such as the insulation of growing room floors, and practical changes, such as new hygiene procedures specifically targeting *L. monocytogenes* on floors and elimination of power‐hose use unless there is a cookout step after its use. In addition, more attention to detail overall in ensuring effective cleaning and sanitation should be given to ensure product safety.

## CONFLICT OF INTEREST

The authors declare no conflict of interest.
